# Supplementary material for: A novel antifolate suppresses growth of FPGS-deficient cells and overcomes methotrexate resistance
Source: Life Sci Alliance. 2023 Aug 17;6(11):e202302058. doi: 10.26508/lsa.202302058 (PMC10435995; doi:10.26508/lsa.202302058)
Supplement: Supplementary file 1 [file LSA-2023-02058_TableS1.doc]

**Supplementary Table 1. C1 inhibits growth of cancer cell lines with variable potency, related to Figure 1.** IC_50_ values for C1 on a panel of cancer cell lines of varying origin. IC_50_ values were determined using Sulforhodamine B viability assay after 72 hours treatment. Z-scores are calculated over all tested cell lines, using log-transformed IC_50_ values.

| **Compound** | **Cell line** | **Origin** | **IC_50_ (M)** | **Z-score** |
| --- | --- | --- | --- | --- |
| C1 | NCIH82 | lung | 8.82E-08 | -1.285 |
| C1 | RDES | bone | 1.19E-07 | -1.2803 |
| C1 | UMUC3 | bladder | 1.04E-07 | -1.1919 |
| C1 | HCT116 | colon | 9.76E-08 | -1.1499 |
| C1 | SKMEL5 | skin | 1.41E-07 | -1.1259 |
| C1 | A375 | skin | 1.07E-07 | -1.1085 |
| C1 | SKHEP1 | liver | 1.12E-07 | -1.104 |
| C1 | MHHES1 | bone | 1.24E-07 | -1.0747 |
| C1 | HL-60 | hematological | 1.15E-07 | -1.0688 |
| C1 | NCIH460 | lung | 1.06E-07 | -1.0676 |
| C1 | A2780 | ovary | 1.20E-07 | -1.033 |
| C1 | MT3 | breast | 1.43E-07 | -1.0062 |
| C1 | HCT15 | colon | 1.33E-07 | -0.9624 |
| C1 | 786O | kidney | 1.37E-07 | -0.9524 |
| C1 | MDAMB468 | breast | 4.91E-07 | -0.9506 |
| C1 | A549 | lung | 1.44E-07 | -0.9497 |
| C1 | MV4-11 | hematological | 1.81E-07 | -0.938 |
| C1 | THP-1 | hematological | 1.67E-07 | -0.9192 |
| C1 | T24 | bladder | 1.54E-07 | -0.918 |
| C1 | SKLMS1 | uterus | 1.64E-07 | -0.9093 |
| C1 | MIAPACA2 | pancreas | 1.65E-07 | -0.8883 |
| C1 | C33A | endometrial | 2.06E-07 | -0.8848 |
| C1 | L-363 | hematological | 1.71E-07 | -0.8753 |
| C1 | JAR | placenta | 1.55E-07 | -0.86 |
| C1 | HT1080 | connective tissue | 1.52E-07 | -0.8422 |
| C1 | SW620 | colon | 1.88E-07 | -0.839 |
| C1 | SU-DHL-6 | hematological | 2.65E-07 | -0.8262 |
| C1 | RAMOS | hematological | 1.59E-07 | -0.8076 |
| C1 | DLD1 | colon | 1.78E-07 | -0.7953 |
| C1 | SKMEL28 | skin | 2.63E-07 | -0.7852 |
| C1 | EJ28 | bladder | 1.72E-07 | -0.7675 |
| C1 | MINO | hematological | 2.47E-07 | -0.7634 |
| C1 | MG63 | bone | 2.39E-07 | -0.7053 |
| C1 | LOVO | colon | 2.62E-07 | -0.6768 |
| C1 | OVCAR4 | ovary | 4.21E-07 | -0.6337 |
| C1 | MDAMB435 | skin | 2.44E-07 | -0.5985 |
| C1 | HT29 | colon | 3.33E-07 | -0.5687 |
| C1 | PANC1 | pancreas | 3.95E-07 | -0.5355 |
| C1 | SKBR3 | breast | 7.89E-07 | -0.5334 |
| C1 | ACHN | kidney | 5.64E-07 | -0.4636 |
| C1 | A431 | skin | 4.26E-07 | -0.4299 |
| C1 | KASUMI-1 | hematological | 4.99E-06 | -0.4286 |
| C1 | HEPG2 | liver | 4.22E-07 | -0.4243 |
| C1 | JIMT1 | breast | 7.04E-07 | -0.3771 |
| C1 | IGROV1 | ovary | 7.14E-07 | -0.3648 |
| C1 | MDAMB436 | breast | - | -0.2949 |
| C1 | CLS439 | bladder | 7.14E-07 | -0.1862 |
| C1 | PLCPRF5 | liver | 5.80E-07 | -0.1766 |
| C1 | DU145 | prostate | 1.39E-06 | 0.0068 |
| C1 | COLO205 | colon | 9.51E-07 | 0.0721 |
| C1 | BXPC3 | pancreas | 2.10E-06 | 0.0855 |
| C1 | NCIH292 | lung | 2.45E-06 | 0.0876 |
| C1 | A673 | muscle | 1.32E-06 | 0.0915 |
| C1 | TE671 | muscle | 9.46E-07 | 0.1128 |
| C1 | PC3 | prostate | 2.14E-06 | 0.1585 |
| C1 | WSU-NHL | hematological | 8.89E-07 | 0.1942 |
| C1 | SKNSH | brain | 4.65E-06 | 0.1985 |
| C1 | NCI-H23 | lung | 1.87E-06 | 0.2372 |
| C1 | ASPC1 | pancreas | 1.37E-06 | 0.2688 |
| C1 | A204 | muscle | 1.71E-06 | 0.3428 |
| C1 | CAKI1 | kidney | 2.13E-06 | 0.3936 |
| C1 | JEG3 | placenta | 1.20E-06 | 0.3991 |
| C1 | CALU6 | lung | 3.86E-06 | 0.5387 |
| C1 | CASKI | endometrial | 2.69E-06 | 0.6131 |
| C1 | OVCAR3 | ovary | 3.22E-06 | 0.6192 |
| C1 | RD | muscle | 5.21E-06 | 0.6516 |
| C1 | MCF7 | breast | 3.27E-06 | 0.6977 |
| C1 | EFO21 | ovary | 8.65E-06 | 0.8272 |
| C1 | 5637 | bladder | 2.73E-06 | 0.8521 |
| C1 | HEK293 | kidney | 3.83E-06 | 0.8724 |
| C1 | 22RV1 | prostate | 2.77E-06 | 0.9083 |
| C1 | SKOV3 | ovary | - | 0.9111 |
| C1 | GRANTA-519 | hematological | 3.95E-06 | 0.9249 |
| C1 | J82 | bladder | 2.79E-06 | 0.9732 |
| C1 | PANC1005 | pancreas | 6.10E-06 | 1.0961 |
| C1 | UO31 | kidney | 4.79E-06 | 1.0962 |
| C1 | CACO2 | colon | 7.00E-06 | 1.1947 |
| C1 | BT20 | breast | - | 1.2169 |
| C1 | SF268 | brain | 9.86E-06 | 1.2301 |
| C1 | SKNAS | brain | - | 1.2744 |
| C1 | NCIH358M | lung | 1.22E-05 | 1.3141 |
| C1 | COLO678 | colon | 5.98E-06 | 1.4093 |
| C1 | SF295 | brain | 7.79E-06 | 1.5228 |
| C1 | MDAMB231 | breast | 1.03E-05 | 1.5422 |
| C1 | IMR90 | lung | - | 1.7451 |
| C1 | U2OS | bone | 1.92E-05 | 1.7557 |
| C1 | SAOS2 | bone | - | 1.7808 |
| C1 | HELA | endometrial | - | 1.8487 |
| C1 | SU-DHL-10 | hematological | - | 2.2091 |
| C1 | U87MG | brain | - | 2.4565 |
| C1 | SNB75 | brain | - | 2.6142 |
| C1 | HS578T | breast | - | - |
| C1 | HS729 | muscle | - | - |
| C1 | PBMC | hematological | - | - |
