# Supplementary material for: A novel antifolate suppresses growth of FPGS-deficient cells and overcomes methotrexate resistance
Source: Life Sci Alliance. 2023 Aug 17;6(11):e202302058. doi: 10.26508/lsa.202302058 (PMC10435995; doi:10.26508/lsa.202302058)
Supplement: Supplementary file 2 [file LSA-2023-02058_TableS2.doc]

**Supplementary Table 2. Stabilized hits and candidate hits of compound C1 identified in LS 174T cells, related to Figure 1D.** Intact LS 174T cells were treated with 10 μM C1 for 1 hour, followed by thermal proteome profiling. The table of all stabilized hits and candidates includes stability scores and false discovery rates (FDR). Destabilized proteins are excluded from this table but are included as source data.

| **Gene name** | **Protein ID** | **Stability score (C1 vs. DMSO)** | **FDR** | **Annotation** |
| --- | --- | --- | --- | --- |
| DHFR | P00374 | 15.10 | 1.15E-14 | hit |
| S100A16 | Q96FQ6 | 4.93 | 3.77E-03 | hit |
| NUDT1 | P36639 | 4.67 | 1.15E-14 | hit |
| HNMT | P50135 | 3.78 | 1.15E-14 | hit |
| LSS-1 | P48449-1 | 3.61 | 1.15E-14 | hit |
| ALDH1B1 | P30837 | 3.28 | 1.15E-14 | hit |
| FAM162A | Q96A26 | 3.21 | 1.81E-03 | hit |
| HMGA1-1 | P17096-1 | 3.11 | 5.57E-04 | hit |
| TYMS | P04818 | 2.97 | 1.15E-14 | candidate |
| TJP1 | Q07157 | 2.94 | 3.36E-08 | candidate |
| LMNB2 | Q03252 | 2.88 | 3.72E-02 | candidate |
| CHMP5 | Q9NZZ3 | 2.75 | 8.51E-06 | candidate |
| DSP-1 | P15924-1 | 2.70 | 3.81E-02 | candidate |
| SQOR | Q9Y6N5 | 2.60 | 1.05E-13 | candidate |
| ESPN-1 | B1AK53-1 | 2.59 | 9.87E-09 | candidate |
| TXNRD2-1 | Q9NNW7-1 | 2.54 | 6.48E-03 | candidate |
| SRA1 | Q9HD15 | 2.53 | 1.33E-02 | candidate |
| TXNRD1-1 | Q16881-1 | 2.47 | 2.11E-03 | candidate |
| DHCR7 | Q9UBM7 | 2.27 | 2.29E-08 | candidate |
| S100A13 | Q99584 | 2.19 | 1.15E-14 | candidate |
| OCLN-1 | Q16625-1 | 2.09 | 1.15E-14 | candidate |
| CFL1 | P23528 | 2.06 | 3.04E-03 | candidate |
| AMPD2-1 | Q01433-1 | 2.00 | 1.15E-14 | candidate |
