# Supplementary material for: A novel antifolate suppresses growth of FPGS-deficient cells and overcomes methotrexate resistance
Source: Life Sci Alliance. 2023 Aug 17;6(11):e202302058. doi: 10.26508/lsa.202302058 (PMC10435995; doi:10.26508/lsa.202302058)
Supplement: Supplementary file 4 [file LSA-2023-02058_TableS3.doc]

**Supplementary Table 3. TMT labels used for thermal proteome profiling.**

| **TMT label** | **Condition** | **Replicate** |
| --- | --- | --- |
| 126 | DMSO | 1 |
| 127N | DMSO | 2 |
| 128C | DMSO | 3 |
| 129N | C1 | 1 |
| 130C | C1 | 2 |
| 131 | C1 | 3 |
